# Allergic diseases of the skin and drug allergies – 2003. Augmented telomerase activity and reduced telomere length as a disease makrer in parthenium induced contact dermatitis patients

**DOI:** 10.1186/1939-4551-6-S1-P93

**Published:** 2013-04-23

**Authors:** Alpana Sharma, Nasim Akhtar, Vivek Anand, Kaushal K Verma

**Affiliations:** 1 Biochemistry, All India Institute of Medical Sciences, New Delhi, India; 2 Dermatology & Venereology, All India Institute of Medical Sciences, New Delhi, India

## Background

*Parthenium dermatitis* is a chronic inflammatory disease with activated T-lymphocytes that recognize the antigens and undergo proliferation and differentiation. T-cells have a pathogenic role in many inflammatory diseases. Telomeres are specialized repeats at the end of chromosomes that protect it from degradation, end-to-end fusion and important for the integrity. Till date there is no report on telomerase activity and TRF length in the lymphocytes of parthenium dermatitis. The aim of our study was to observe the involvement of T_H_1 & T_H_2 type responses and to measure telomerase activity and telomere length in PBMC, CD4^+^ and CD8^+^T lymphocytes in parthenium dermatitis patients.

## Methods

The study cohort consists of 50 patients of parthenium dermatitis confirmed by patch testing, 22 follow up cases in remission and 40 age matched healthy controls. T_H_1 (IL-2 & IFN-γ) and T_H_2 (IL-4 & IL-10) cytokines were measured by ELISA. Telomerase activity was measured by telomere repeat amplification protocol by PCR-ELISA and telomere length by *T*elo *TAGGG*Telomere Length Assay Kit.

## Results

The mean concentration of T_H_1 cytokines were increased significantly (p < 0.001) as compared to controls whereas it was decreased in case of T_H_2. In follow up remission cases levels of T_H_1 cytokines were significantly (p < 0.05) decreased but change in T_H_2 cytokines level were insignificant (p > 0.05) when compared with untreated cases. Significantly (p < 0.05) elevated levels of telomerase activity and reduced telomere length was observed in PBMC, CD4^+^ and CD8^+^T cells of parthenium dermatitis patients as compared to healthy individuals. In post-treatment remission cases mean telomerase activity was significantly (p < 0.05) reduced whereas change in telomere length was insignificant, as compared to pre-treatment cases.

## Conclusions

The higher concentration of T_H_ cytokines strengthens the hypothesis of chronic stimulation of T cells in this inflammatory disease. Elevated telomerase activity and reduced TRF length might support the understanding of mechanisms in pathogenesis of parthenium dermatitis that are characterized by the recruitment of T lymphocytes. The augmented telomerase activity in pre-treatment cases and reduced activity in case of remission signifies that this might be established as a potential diagnostic/prognostic marker for parthenium dermatitis in future.

